# Measuring the neurodevelopmental trajectory of excitatory-inhibitory balance via visual gamma oscillations

**DOI:** 10.1162/imag_a_00527

**Published:** 2025-04-06

**Authors:** Natalie Rhodes, Lukas Rier, Krish D. Singh, Julie Sato, Marlee M. Vandewouw, Niall Holmes, Elena Boto, Ryan M. Hill, Molly Rea, Margot J. Taylor, Matthew J. Brookes

**Affiliations:** Sir Peter Mansfield Imaging Centre, School of Physics and Astronomy, University of Nottingham, Nottingham, United Kingdom; Diagnostic & Interventional Radiology, The Hospital for Sick Children, Toronto, Canada; Program in Neurosciences & Mental Health, SickKids Research Institute, Toronto, Canada; Cerca Magnetics Ltd., Nottingham, United Kingdom; Cardiff University Brain Research Imaging Centre, School of Psychology, Cardiff, United Kingdom; Autism Research Centre, Bloorview Research Institute, Holland Bloorview Kids Rehabilitation Hospital, Toronto, Canada; Department of Medical Imaging, University of Toronto, Toronto, Canada

**Keywords:** gamma oscillations, excitation-inhibition balance, neurodevelopment, magnetoencephalography, optically pumped magnetometers

## Abstract

Disruption of the balance between excitatory and inhibitory neurotransmission (E-I balance) is thought to underlie many neurodevelopmental disorders; however, its study is typically restricted to adults, animal models, and the lab-bench. Neurophysiological oscillations in the gamma frequency band relate closely to E-I balance, and a new technology—OPM-MEG—offers the possibility to measure such signals across the lifespan. We used OPM-MEG to measure gamma oscillations induced by visual stimulation in 101 participants, aged 2–34 years. We demonstrate a significantly changing spectrum with age, with low-amplitude broadband gamma oscillations in children and high-amplitude band limited oscillations in adults. We used a canonical cortical microcircuit to model these signals, revealing a significant decrease in the ratio of excitatory to inhibitory signalling with age in the superficial pyramidal neurons of the visual cortex. Our findings detail the first MEG metrics of gamma oscillations and their underlying generators from toddlerhood, providing a benchmark against which future studies can contextualise.

## 1 Introduction

The maintenance of a balance between excitatory and inhibitory neurotransmission (E-I balance) is essential for healthy brain function and its disruption underlies a range of psychiatric conditions, notably autistic spectrum disorder (ASD) (Nelson & Valakh, 2015;Rubenstein & Merzenich, 2003;Sohal & Rubenstein, 2019). High-frequency neurophysiological oscillations in the gamma range (>30 Hz) play a key role in information processing (Fernandez-Ruiz et al., 2023) and arise due to interactions between neuronal excitation and inhibition (Bartos et al., 2007;Vinck et al., 2013). Thus, measurement of gamma oscillations can provide a powerful metric of E-I balance (Gray & Singer, 1989;Gray et al., 1989;Whittington et al., 1995). Despite this importance, our understanding of gamma oscillations, their developmental trajectory in early childhood and perturbation by disorders remains poorly characterised, and this is largely due to instrument limitations. Here, we use a new neurophysiological imaging platform to measure gamma oscillations in individuals from early childhood to adulthood and a model of neural circuitry to investigate their underlying neural generators.

Gamma oscillations can be measured non-invasively using either electro- or magnetoencephalography (EEG or MEG), with MEG providing more robust data. However, both techniques have limitations, particularly for children. In EEG, the gamma signal (which manifests as an electrical potential difference across the scalp surface) is diminished in amplitude and distorted spatially by the skull (Baillet, 2017). EEG signals are also obfuscated by interference generated by non-neural sources such as muscles (Boto et al., 2019;Muthukumaraswamy, 2013), making it difficult to measure gamma reliably, particularly if subjects move (which is common with children). MEG, which measures magnetic fields generated by neural currents, is less affected by non-neural artefacts and has better spatial specificity than EEG (because magnetic fields are less distorted by the skull than electrical potentials). This means that gamma oscillations have a higher signal-to-noise ratio (SNR) and their origin can be better localised when using MEG rather than EEG (Muthukumaraswamy & Singh, 2013). Multiple studies argue that MEG is the measurement of choice for gamma oscillations (Gaetz et al., 2011;Hall et al., 2005;Muthukumaraswamy et al., 2009,^2010^;Orekhova et al., 2015;Takesaki et al., 2016;Tan et al., 2016). However, MEG systems classically rely on cryogenically cooled sensors that are fixed in position in a one-size-fits-all helmet. Such systems cannot cope with changing head size through childhood or large subject motion relative to the static sensors. Consequently, most extant MEG studies of gamma oscillations are limited to adults.

As ASD has a typical diagnostic age of 3 years and above, if we are to understand its neural substrates, E-I imbalance (and gamma oscillations) must be measured reliably in children from 2–3 years of age and upward. While this is challenging using conventional MEG equipment, new technology, based on optically pumped magnetometers (OPMs) (for a review seeSchofield et al. (2023)), shows significant promise. OPMs uniquely allow MEG signals to be recorded using small (Lego-brick-sized) sensors mounted in wearable helmets (Boto et al., 2018;Hill et al., 2020), which adapt to different head sizes and allow for movement during scanning. This provides an ideal environment to gather high-fidelity data in children, and studies have already shown that OPM-MEG can be used to measure neurophysiological signals in the early years of life (Corvilain et al., 2025;Hill et al., 2019) and can assess neurodevelopmental changes in neurophysiology (Rier et al., 2024;Vandewouw et al., 2024). This platform, therefore, offers the best opportunity for measurement of gamma oscillations, and subsequent modelling of underlying neural circuitry to understand how E-I balance changes with age.

Here, we characterised the neurodevelopmental trajectory of gamma oscillations from age 2 years to adulthood in a cohort of >100 participants. We used a newly developed child-friendly OPM-MEG system to collect data during a visual task that is known to elicit gamma oscillations in the primary visual cortex (Hall et al., 2005). These visual gamma effects have been associated with feature integration (Eckhorn et al., 1988;Gray et al., 1989), object representation (Tallon-Baudry & Bertrand, 1999), and selective attention (Fell et al., 2003). Existing studies suggest that features of these oscillations, such as peak frequency and relative amplitude, are different in children relative to adults (Gaetz et al., 2011;Orekhova et al., 2018) (albeit in older children), in ASD (Orekhova et al., 2023;Safar et al., 2021), and twin studies suggest they are highly heritable (Pelt et al., 2012). The cellular generators of visual gamma oscillations have been described (Spaak et al., 2012;Xing et al., 2012) by modelling the interaction between superficial pyramidal cells and inhibitory interneurons within V1. Having measured gamma oscillations using OPM-MEG, we subsequently employ a dynamic causal model (DCM)—based on a canonical cellular microcircuit (Shaw et al., 2017)—to investigate the contributions of inhibitory and excitatory neurotransmission to the gamma signal. We hypothesised that OPM measurement of gamma oscillations alongside DCM would demonstrate an E-I balance change in the superficial layer of V1 as the human brain matures.

## 2 Methods

OPM-MEG data were collected using two systems: one located at the Sir Peter Mansfield Imaging Centre, University of Nottingham, UK (UoN), one located at The Hospital for Sick Children (SickKids), Toronto, Canada (SK).

### 2.1 Participants and paradigm

The study was approved by the local research ethics board committee at both sites. All adult participants provided written informed consent. A legal guardian for all participants under 18 years provided the written informed consent, and the child gave verbal assent. The study included 102 typically developing participants (aged 2–34 years; 44 male; seeTable S2in Supplementary Information). At UoN, 27 children and 26 adults were scanned; 24 children and 26 adults were scanned at SK. Children were always accompanied by a parent and at least one experimenter inside the magnetically shielded room (MSR). Adult data were sex- and age-matched across the two sites to enable a cross-site comparison.

**Fig. 1.**
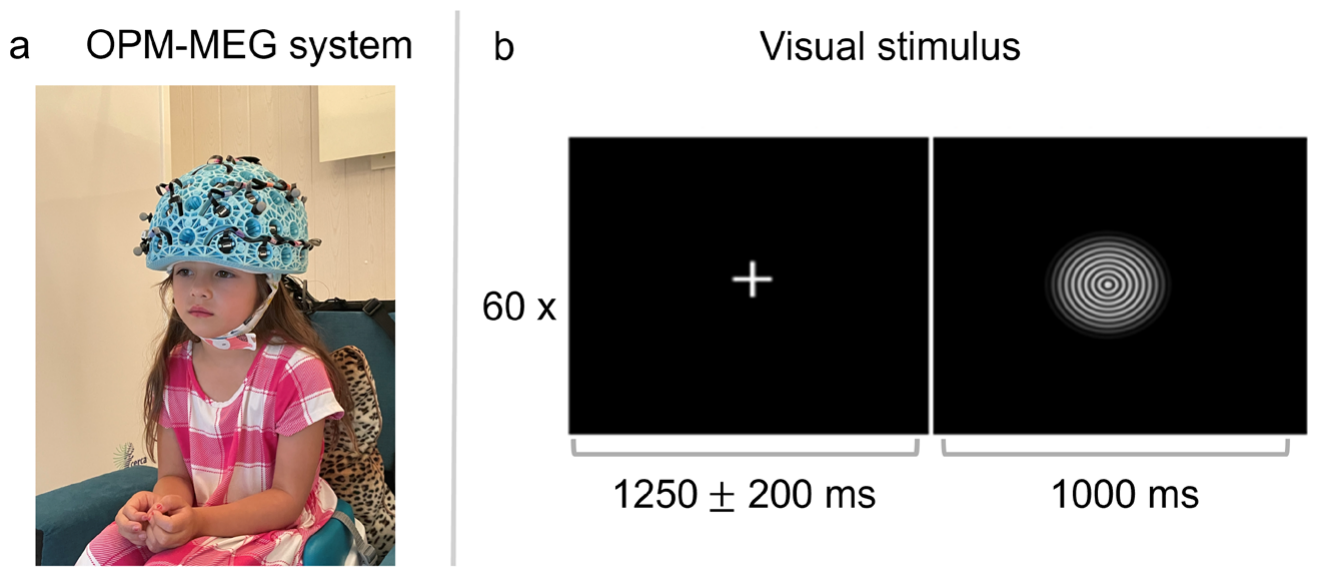
Methods. (a) An image of a child in the OPM-MEG system, (b) the concentric circles visual stimulus and paradigm timing, which was presented for 60 trials. Parental consent and authorization for publication of the image of the participant has been obtained.

Visual stimulation comprised an inwardly moving circular grating moving at$1.2{}^{\circ}s^{-1}$(Fig. 1b). The grating was displayed centrally at 100% contrast and subtended a visual angle of 7.6°, with 1.32 cycles per degree. A single trial comprised 1000 ms of stimulation followed by a jittered rest period with a white fixation cross located centrally on a black screen for 1250 ± 200 ms. Sixty trials in total were shown and these circles trials were interspersed with images of faces (data not included). Precise timing of the onset and offset of stimulation was sent from the stimulus PC to the OPM-MEG system via a parallel port.

### 2.2 Data acquisition

The UoN OPM array comprised 64 triaxial OPMs (3^rd^generation QZFM; QuSpin, Colorado, USA), enabling up to 192 channels of magnetic field measurement. The SK system comprised 40 dual-axis OPMs (3^rd-^generation QZFM; QuSpin), enabling up to 80 channels of magnetic field measurement. The two systems had a similar design (Cerca Magnetics Ltd. Nottingham, UK), and channels were located to ensure good coverage of the visual cortices (see also Supplementary InformationTable S1; equivalence between systems is shown inFig. S1).

In both systems, sensors were combined to form an array and integrated with other hardware (e.g., for magnetic field control) and software (e.g., for stimulus delivery and data acquisition) to form complete neuroimaging systems (Cerca Magnetics Ltd, Nottingham UK). Sensors were mounted in rigid 3D-printed helmets (five sizes were available). Participants wore a thin aerogel cap or had insulating padding under the helmet for thermal insulation. Participants were seated in a patient support at the centre of the MSR. The UoN system was housed in an OPM-optimised MSR which comprises four layers of mu-metal, one layer of copper, and is equipped with degaussing coils. The SK system was housed in a repurposed MSR from a cryogenic-MEG system which comprised two layers of mu-metal and one layer of aluminium (Vacuumschmelze, Hanau, Germany). In both systems, bi-planar coils (Cerca Magnetics Ltd.) surrounded the participants to provide active magnetic field control (Holmes et al., 2018). In the UoN system, coil currents were applied to cancel out the residual (temporally static) magnetic field (Rea et al., 2022;Rhodes et al., 2023;Rier et al., 2024). At SK (where time-varying field shifts were larger), a reference array provided dynamic measurement of the environmental magnetic field and feedback to the bi-planar coils enabled real-time compensation of both static and dynamic magnetic field changes (Holmes et al., 2019). Equivalent data from these two systems have been demonstrated previously (Hill et al., 2022). In both systems, participants were free to move throughout data acquisition (but were not encouraged to do so). Data were collected at a sampling rate of 1200 Hz, from all sensors, using a National Instruments (NI, Texas, US) data acquisition system interfaced with LabView (NI).

For coregistration of sensor geometry to brain anatomy, two 3D digitisations of the participant’s head (with and without the OPM helmet) were acquired using a structured light camera (Einscan H, SHINING 3D, Hangzhou, China). These digitisations, coupled with accurate knowledge of the helmet structure from its computer aided design, allowed identification of the sensor locations/orientations relative to the head. They also enabled generation of a ‘pseudo-MRI’ which provided an approximation of the underlying brain anatomy (for more details seeRhodes et al., 2025). Briefly, age-matched template MRIs (Richards et al., 2016) were warped to the individual participant’s 3D head digitisation using FSL FLIRT (Jenkinson et al., 2002). For some of the youngest participants, head digitisation without the helmet (which is only required for the pseudo-MRI generation) failed or was not acquired (n = 20) and the age-matched templates were used as the pseudo-MRI without warping.

### 2.3 Data analyses

Data processing was identical at both sites and implemented using custom pipelines (https://github.com/nsrhodes/gamma_opm_2024). Bad channels (those that either had high noise or low signal) were identified by manual inspection of the channel power spectra and removed. Data were notch filtered at the powerline frequency (50 Hz for UoN and 60 Hz for SK) and 2 harmonics. A 1–150 Hz band pass filter was applied, following which data were epoched to 3 s trials encompassing 1 s prior to the onset of the circle and 2 s after. Bad trials were identified as those with trial variance greater than 3 standard deviations from the mean and were removed. Visual inspection was carried out, and any further trials with noticeable artefacts were removed. ICA was used to remove eye blink and cardiac artefacts (implemented in FieldTrip (Oostenveld et al., 2011)), and homogeneous field correction (HFC) was applied to reduce interference that manifests as a spatially homogeneous field (Tierney et al., 2021). Following data pre-processing, one child participant was removed due to failure to acquire a complete 3D head digitisation with the helmet on (necessary for accurate coregistration). We removed 13±9 (mean±standard deviation) trials in children and 7±4 trials in adults due to excessive interference. Trials were then matched across age groups by selecting and removing additional trials in adults and older children, and this resulted in each age group having an average of 43 trials. On average, we had 159±11 (mean±standard deviation) channels of data at UoN, and 78±3 channels at SK.

We used an LCMV beamformer to project magnetic fields recorded at the sensors into estimates of current dipole strength in the brain (Van Veen et al., 1997). The forward model was constructed using a single-shell model (Nolte, 2003), fitted to the pseudo-MRI, and implemented in FieldTrip (Oostenveld et al., 2011). Voxels were placed on an isotropic 4 mm grid covering the whole brain, and an additional 1 mm isotropic grid covering the visual cortex (identified by dilating a mask of the left and right cuneus from the AAL atlas (Hillebrand et al., 2016;Tzourio-Mazoyer et al., 2002) with a 5 mm spherical structuring element). Covariance matrices were generated using 1–150 Hz broadband data spanning all trials (excluding bad trials), regularized using the Tikhonov method with a regularization parameter of 5% of the maximum eigenvalue of the unregularized matrix (Brookes et al., 2008). This matrix was used to compute the beamformer weighting parameters used for all subsequent calculations.

Pseudo-T statistical images were constructed by contrasting either alpha or gamma power during stimulation and rest. Specifically, we derived four additional covariance matrices ($C_{ON\text{​}\_\text{​}alpha}$,$C_{OFF\text{​}\_alpha}$,$C_{ON\text{​}\_gamma}$and$C_{OFF\text{​}\_gamma}$). For the gamma matrices, we used 30–80 Hz filtered data and for alpha band we used 6–14 Hz filtered data. The ON window was 0.3–1 s, and the OFF window was -0.8 to -0.1 s (timings relative to the onset of the circle.

Time frequency spectra (TFS) showing neurophysiological activity at the locations of maximum gamma/alpha modulation (identified using the 1 mm resolution images) were derived. TFS data in the 1–100 Hz frequency range were generated by first sequentially filtering broadband beamformer projected data into 45 overlapping frequency bands (2 Hz separation, 4 Hz bandwidth). For each band, the Hilbert transform was computed to give the analytic signal; the absolute value was computed to derive a measure of instantaneous oscillatory amplitude, and these Hilbert envelopes were averaged across trials and concatenated in the frequency dimension. For each band, a mean baseline amplitude was taken in the -0.8 s to -0.1 s window and subtracted. Data were then normalised by the baseline values to give a measure of relative change in amplitude. These data were collapsed in time to give spectral relative change (i.e.,Figs. 3and5). In all cases, we investigated the statistical relations between age and amplitude modulation using Spearman’s correlation.

#### 2.2.1 DCM

Neurophysiologically informed modelling was performed using dynamic causal modelling (DCM) for steady-state responses implemented in SPM8 (Moran et al., 2009;Shaw et al., 2017). The canonical microcircuit structure (shown inFig. 4a) describes a model that strikes a balance between biological reality and complexity that can be modelled. The model estimates membrane potentials and postsynaptic currents of cell populations across four interacting cortical layers through differential equations. We followed the methods described inShaw et al. (2017). Briefly, the model takes the spectral content from the time course at the location of maximum gamma modulation, pre-whitens the data to flatten the spectra to reveal alpha, beta, and gamma peaks, and scales the amplitude to ensure the individual outputs are all in the same range. The alpha peak is then explicitly modelled using a single Gaussian (constrained to 8 to 13 Hz) and removed, as the model is capable of generating clear beta and gamma peaks but alpha is thought to be generated over more extensive circuity, including thalamo-cortical interactions (Bastos et al., 2014). Priors are set by first fitting the model to the mean spectral density across all participants (seen inFig. 4b). Finally, the model with the set priors is fit to each individual participant’s spectral signal.

Here, we differ from the analysis described inShaw et al. (2017)by using relative spectra as the model input rather than pre-whitening by removal of the 1/f profile to remove the strong power-law that dominates the signal, as this proved advantageous for OPM data where absolute spectra are more prone to noise (see alsoFig. S2andSection 4). Relative broadband spectra from the beamformer estimated time series at the peak gamma modulation were calculated by taking the power spectral density (PSD) of data during the stimulus (0.3–1 s) minus the PSD of data during the rest (-0.8 to -0.1 s) windows, divided by the rest period. The absolute of these values was derived (so all features are shown as positive peaks). The relative spectra were normalised such that the area under the global average equals 1, but relative peak height was preserved, and the alpha peak was removed as described above. Model priors and parameters that have little or no effect (G1, G3, G10, and G13) are held constant prior to submitting data to model inference as in prior work (Shaw et al., 2017). These processes allow the DCM to estimate the ‘G parameters’ (the model output) that describe the relative contributions of excitatory and inhibitory signals that result in the measured beta and gamma responses, alongside the F-statistic, which represents the log model evidence (a measure of model fit with a complexity penalty). The F-statistic allows for Bayesian Model Comparison, although this was not explored here as we are interested in intersubject variations rather than model selection. Having fitted the model to each subject’s spectrum, we used Spearman’s correlation to investigate the relationship between age and all model parameters. We also investigated the ratio between parameters in the superficial layer (G12/G11) and the deep layer of pyramidal neurons (G6/G9) to probe age changes in the hypothesised E-I balance (Shaw et al., 2017).

## 3 Results

### 3.1 Gamma oscillations change with age

**Fig. 2.**
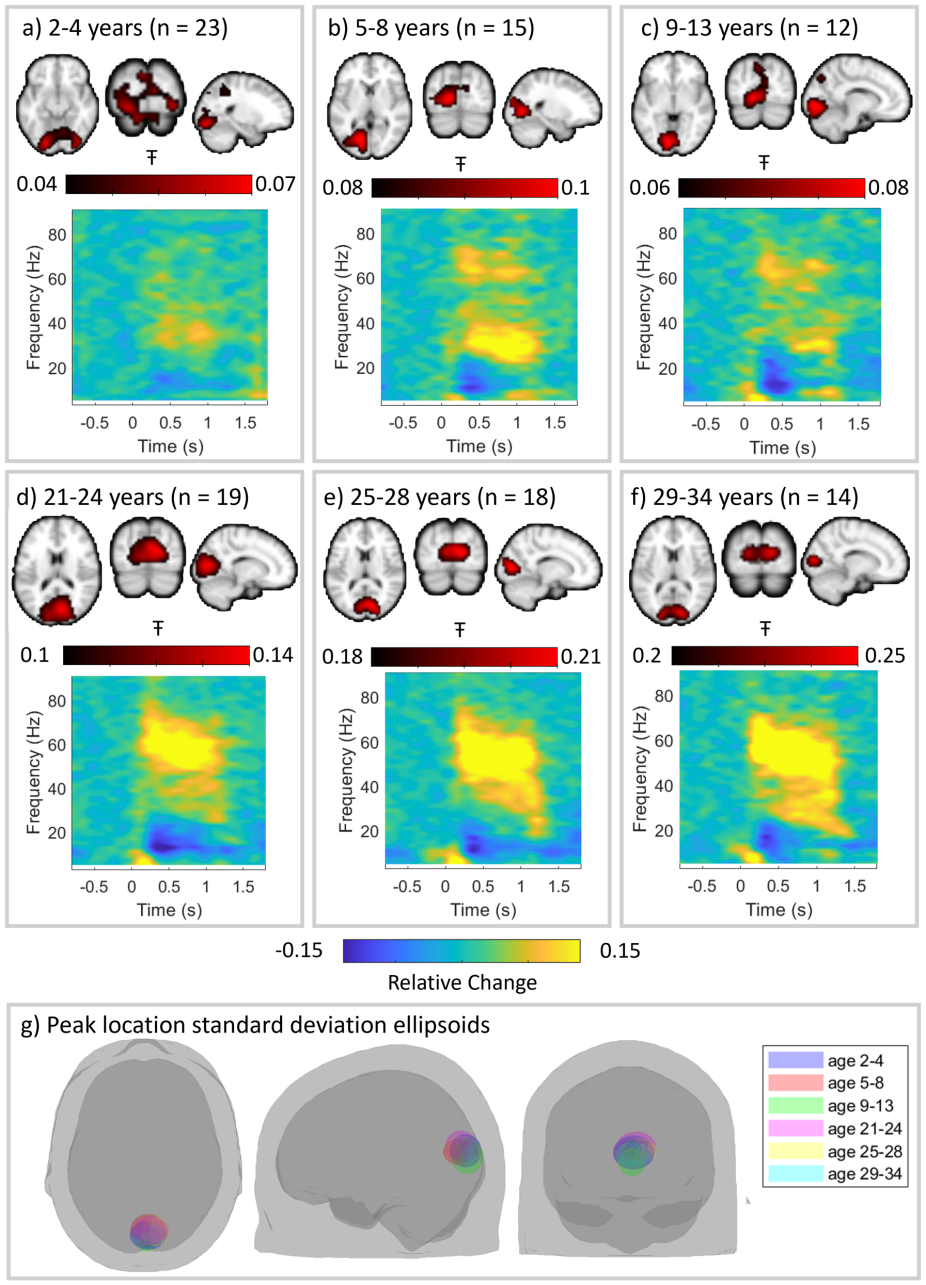
Age-group-specific time-frequency spectrograms show development of gamma oscillations. Participant-averaged pseudo-T statistical images of gamma modulation are shown in red (4 mm resolution) overlaid on the standard brain. The time-frequency spectrograms show group-averaged oscillatory dynamics from the location of largest gamma modulation in the visual cortex. (a) 2–4-year-olds (n = 23), (b) 5–8-year-olds (n = 15), (c) 9–13-year-olds (n = 12), (d) 21–24-year-olds (n = 19), (e) 25–28-year-olds (n = 18), and (f) 29–34-year-olds (n = 14) (ages are inclusive). Note the evolution of spectral signature with age. (g) Ellipsoids describing the mean and standard deviation of the coordinates of the largest gamma modulation for all age groups. We saw no significant difference in the location of the visual gamma response with age in any axis (p = 0.44, p = 0.52, and p = 0.51 for x, y, and z axes, measured using Spearman correlation to test for a systematic shift in spatial localisation due to age).

Figure 2a-f, show the spatial and spectro-temporal signatures of gamma activity for all participants. Data were separated into six age groups and, for all groups, an image showing the spatial distribution of gamma modulation is shown (as a red overlay on the standard brain, averaged across subjects). TFS extracted from the location of peak gamma modulation are also shown. In the TFS, yellow indicates a task-induced increase in oscillatory amplitude relative to baseline, whereas blue indicates a decrease. All age groups showed a peak gamma response that localised to the primary visual cortex, as expected. We saw no significant difference in the location of the visual gamma response with age (seeFig. 2 g) in any axis.

We did, however, see a changing spectro-temporal picture with age. In younger subjects, we saw a task-induced broadband gamma increase (this is also clear in task and rest PSD plots given inFig. S2). In older children, the broadband response remains, and we also observed bimodal gamma activity, most prominent at around 35 Hz and 70 Hz, with the higher-frequency component qualitatively in agreement with the literature in older children (Gaetz et al., 2011;Orekhova et al., 2018). This further evolved to a broad band response with additional high-amplitude narrow band activity at around 60 Hz in adults, consistent with the literature in adults (Bharmauria et al., 2016;Murty et al., 2018;Ray & Maunsell, 2011).

**Fig. 3.**
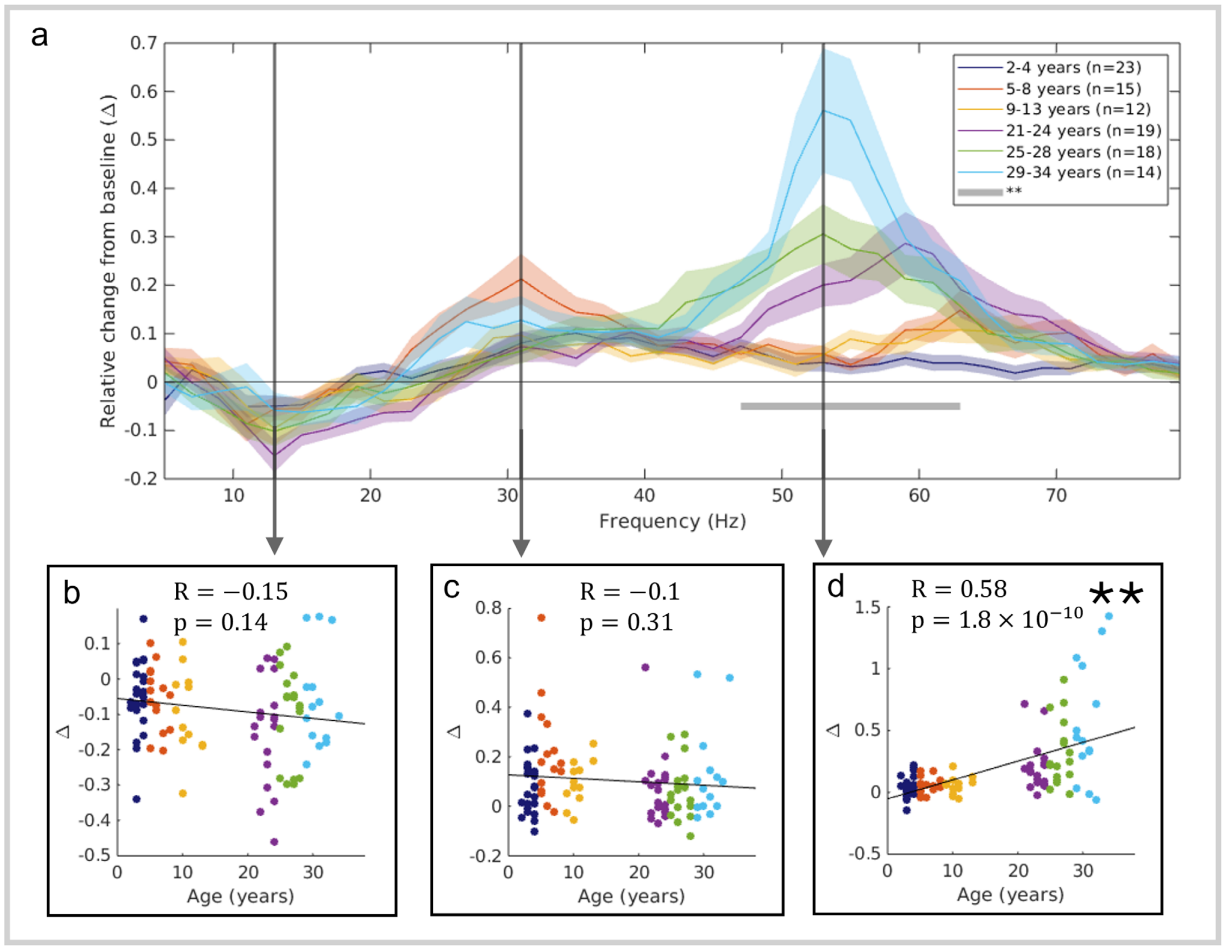
Gamma amplitude changes with age. The stimulus-induced relative change in oscillatory amplitude from baseline is plotted against frequency for the 6 age groups (a). The relative change was measured in the 0.3 to 1 s window post-stimulus compared to the -0.8 s to -0.1 s baseline period (i.e., ((stimulation–baseline)/baseline) for each frequency band). Lines show the group means with shading representing standard error across subjects. The inset scatter plots (b, c, and d) show relative change for all individuals in the study plotted against age (colour indicating age group), with straight lines fitted to the data. We show data in the frequency ranges 11–15 Hz (b) (R=−0.15, p=0.14); 29–33 Hz (c) (R=-0.1, p = 0.31); and 51–55 Hz (d) ($\text{R}=0.58,\text{ p}=1.8\times 10^{-10}$) (all p-values generated using Spearman’s correlation). The star (**) and grey horizontal bar between 45 and 65 Hz indicates significance following Bonferroni correction with a threshold of p < 0.0011 to account for 44 comparisons across different frequency bands.

Figure 3formalises the data inFigure 2by demonstrating statistical significance of the observed spectral changes. The central graph shows stimulus-induced relative change in oscillatory amplitude for the 6 age groups, plotted against frequency. This was calculated by contrasting the 0.3–1 s window (during stimulation) to the -0.8 to -0.1 s (baseline) window (Campbell et al., 2014). The inset plots show relative change in oscillatory amplitude for individual participants, for frequency bands 11–15 Hz, 29–33 Hz, and 51–55 Hz. Here, each data point represents a single individual in the study and data are plotted against age. Spearman’s correlation showed a significant increase in spectral amplitude across gamma frequencies spanning 45–65 Hz (indicated by the grey horizontal bar), peaking in the 51–55 Hz range ($\text{R}=0.58,p=1.8\times 10^{-10}$). There was no significant effect, however, at 11–15 Hz (alpha frequency range) or 29–33 Hz (low gamma) (R=−0.15,p=0.14andR=−0.1,p=0.31, respectively). Separate analysis of the child and adult groups showed trends of positive relation with age across the gamma band (Spearman’s correlation of p < 0.05 in range 59–71 Hz in children and 43–57 Hz in adults), though these did not survive correction for multiple comparisons. This is consistent with a stimulus-induced broadband gamma increase at all ages, demonstrated by the visual localisation and positive relative change, with emergent narrowband effects in adults.

### 3.2 DCM suggests E-I balance drives spectral changes

**Fig. 4.**
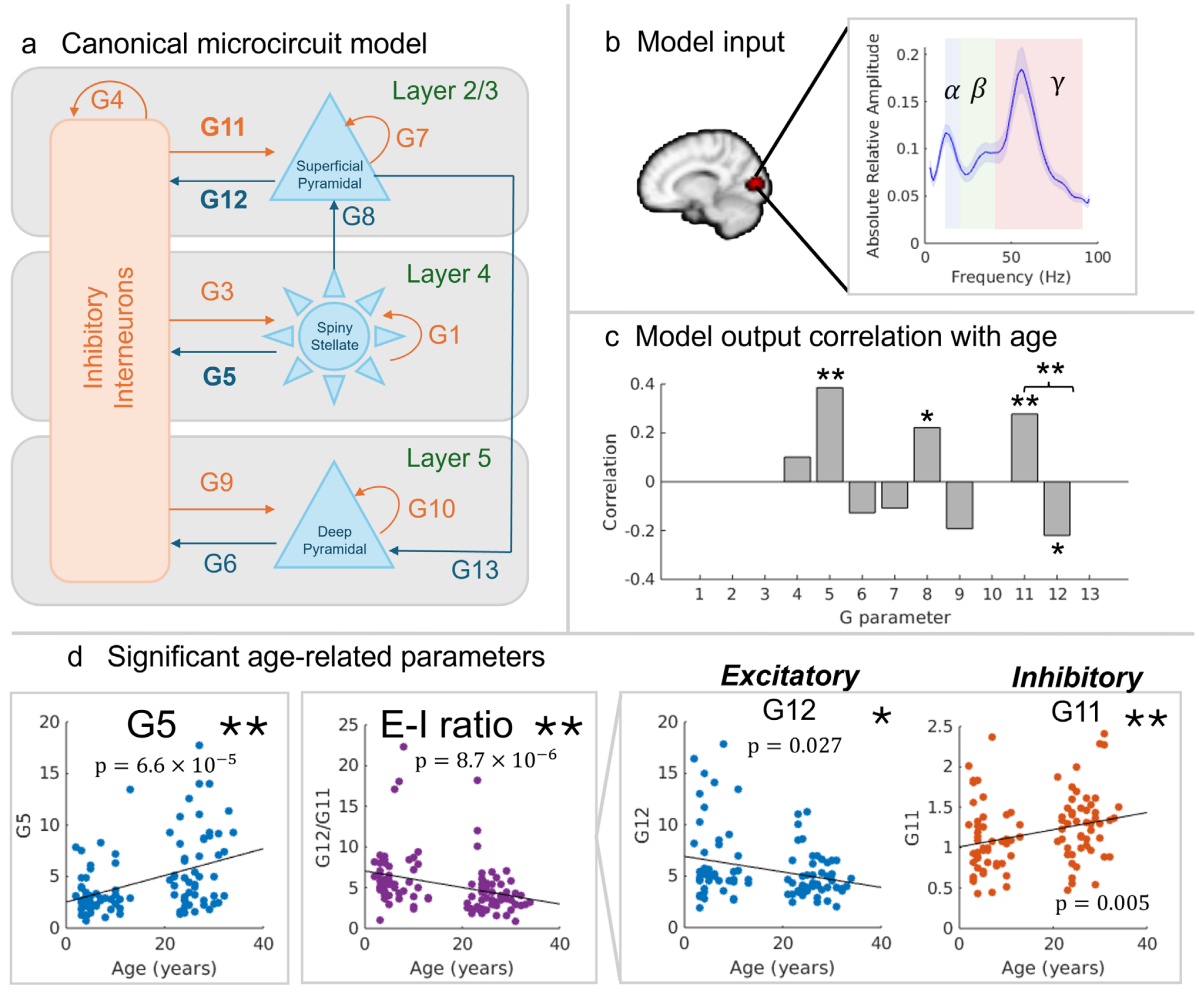
DCM suggests E-I balance underlies age related spectral differences. (a) The canonical microcircuit model describes the relative contribution of cells within the cellular column. The model takes spectral input from data in the visual cortex and fits a set of parameters (G1–G12) which describe the relative contribution of the different neuronal assemblies to the measured signal. Excitatory signals are indicated by blue and inhibitory in orange. (b) The absolute values of the average (across all subjects) relative difference spectrum between active and control windows (divided by the control window), with canonical frequency bands highlighted (alpha in blue, beta in green, and gamma in red). (c) Correlation of the model-derived G parameters with age. Significant age-relations were observed in G5, G11 and the ratio of parameters G12 and G11. (d) Scatter plots for G5 (excitatory); the E-I ratio of G12 and G11, and G12 (excitatory) and G11 (inhibitory) individually. The star (*) indicates uncorrected significance (p < 0.05), and (**) indicates significance following Bonferroni correction with a threshold of p < 0.005 to account for 10 comparisons across parameters.

A local spectral DCM, optimised for V1 (Shaw et al., 2017), was used to determine how inhibitory and excitatory activity drives the observed changes in gamma oscillations between children and adults. This model, which is summarised byFigure 4a, has been verified in recent literature using adult MEG recordings and pharmacological intervention (Shaw et al., 2017,^2020^).Figure 4bshows the average (absolute) relative difference spectrum (between stimulation and rest, divided by baseline) for all participants, highlighting the gamma change, while also showing features of the signal that fall into the alpha and beta bands. Similar spectra (for individuals) were used to fit the DCM. The model output comprised ‘G parameters’, which are related to spectral features as outlined inTable S3in Supplementary Information, and the F-statistic, a metric of model quality of fit, which showed no significant age relation (Fig. S4).Figure 4cshows the results of our correlational analyses between each model parameter and age, with significant relations in parameters G5 (describing the excitatory output from spiny stellate cells to inhibitory interneurons), G11 (the inhibitory connection between inter-neurons and superficial pyramidal neurons) and the ratio between G12 and G11 (which represents the relation between excitatory and inhibitory connections between superficial pyramidal neurons and inhibitory inter-neurons). These relationships remain significant following correction for multiple comparisons and are detailed inTable S4in the Supplementary Information. The parameters demonstrating significant age-related correlations are shown in the scatter plots inFigure 4d; notice that inhibition tends to increase, while excitation decreases in the superficial layer, such that the ratio of excitation to inhibition decreases with increasing age. Spearman’s correlational analysis within the child and adult age groups separately observed the same negative trend in the E-I ratio, although these did not reach significance independently. We independently assessed the G12/G11 ratio for male and female participants, with results presented inFigure S5of the Supplementary Information. Analyses showed that the significant negative relation of E-I balance with age held in both sexes.

### 3.3 Alpha suppression is comparable across ages

**Fig. 5.**
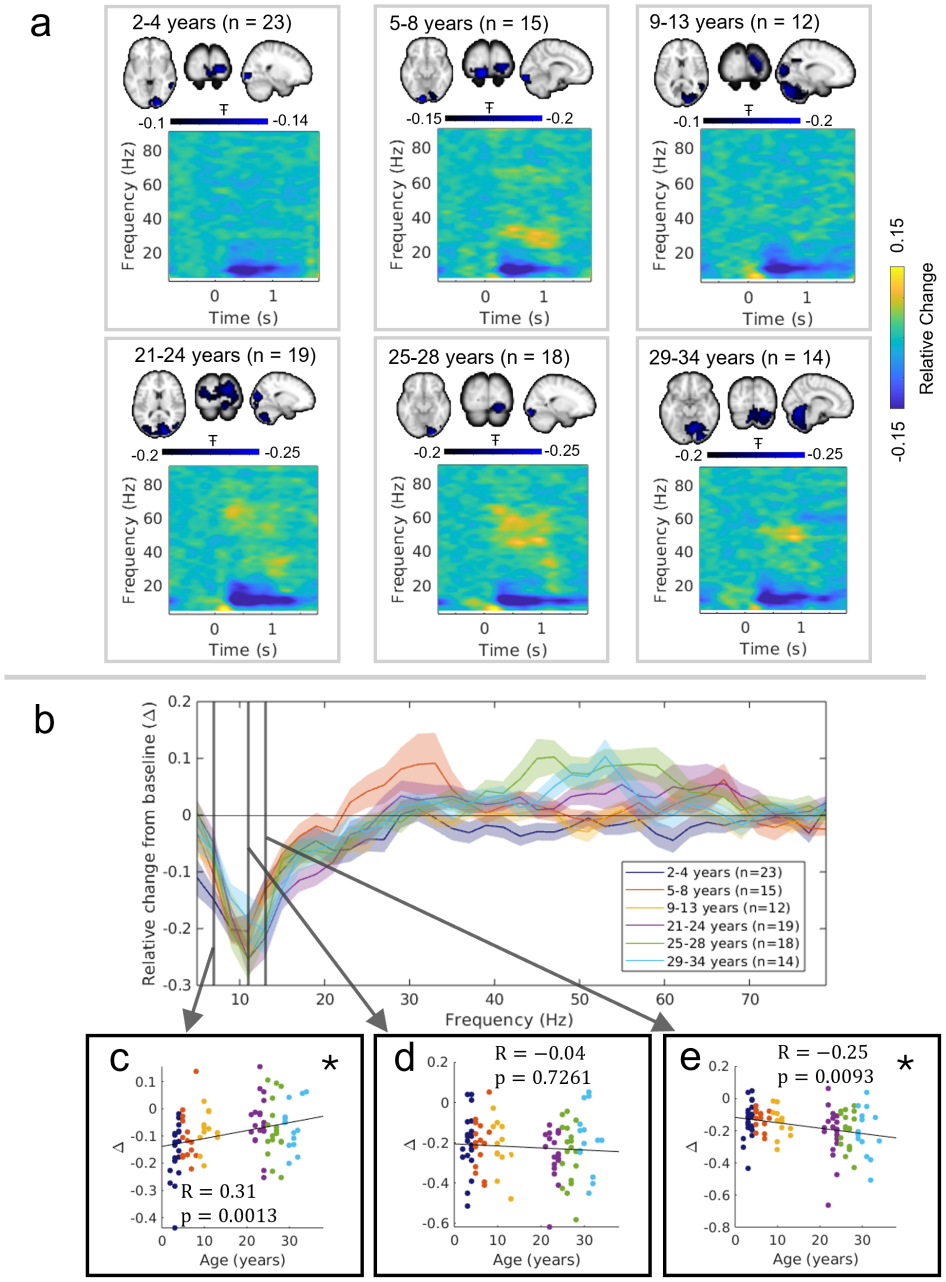
Alpha suppression is comparable across ages. (a) Pseudo-T statistical maps and time-frequency spectrograms from the locations of peak of alpha suppression. Data are divided by age group. (b) Relative change in oscillatory amplitude as a function of frequency (i.e., ((stimulation – baseline)/baseline) for each frequency band). The inset scatter plots show how stimulus-induced amplitude change differs for individuals in the (c) 5–9 Hz range (R=0.31, p=0.0013), (d) 9–3 Hz range (R=−0.04, p=0.7261and (e) 11–15 Hz range (R=−0.25 p=0.0093)bands (colour indicating age group). The star (*) indicates uncorrected significance (p < 0.05).

Finally, for completeness, we assessed how age affects stimulus-induced change of alpha oscillations.Figure 5ashows the spatial signature of alpha suppression (in blue, overlaid on the standard brain) alongside the TFS data from the locations of largest task-induced alpha modulation, across the age groups. Note that these regions differ from those of maximum gamma change (as would be expected from previous studies (Muthukumaraswamy & Singh, 2013)) and, consequently, the gamma change is less prominent. We found that the localisation of the alpha desynchronisation is somewhat lateralised; this was expected based on previous studies (e.g.,Wiesman et al., 2021). We show in our TFS that alpha modulation is clear in all age groups.

InFigure 5b, the spectrum shows relative change in oscillatory amplitude from baseline as a function of frequency (including a zoomed in area over the alpha band). The inset scatter plots show relative change, for individual participants, for the frequency bands 5–9 Hz, 9–13 Hz, and 11–15 Hz. We found no change in alpha modulation for the 9–13 Hz canonical alpha band. However, we saw increased (more negative) 5–9 Hz modulation in younger participants (which was also observed with Spearman’s correlation for only the child participants with p < 0.05) and increased 11–15 Hz modulation for older participants (though these were non-significant following correction for multiple comparisons across 44 frequency bands). This is in broad agreement with the widespread finding that the alpha rhythm’s peak frequency increases with age (Miskovic et al., 2015). We support this finding further by directly assessing the relation between the peak alpha frequency with age, showing a significant positive correlation in Supplementary Information (Fig. S3).

## 4 Discussion

E-I balance (or imbalance) underpins healthy (and atypical) brain function and its characterisation could provide valuable insights into neurodevelopmental disorders (Sohal & Rubenstein, 2019). While in-vitro and animal studies form the basis of such models, the ability to non-invasively characterise E-I balance using imaging offers a means to bridge the gap between experimental animal and in-vivo human physiology. A significant body of literature suggests that gamma oscillations provide a window on E-I balance. For example, animal studies show that visual gamma frequency is reduced by administration of thiopental, which interacts with GABA neurotransmission (Oke et al., 2010). In humans, alcohol, propofol, and ketamine have all been shown to alter gamma amplitude and frequency, which has been attributed to modulation of GABA receptors (Campbell et al., 2014;Saxena et al., 2013;Shaw et al., 2015). These direct pharmacological manipulations suggest that gamma oscillations change with modulation of E-I balance. However, the formation of gamma oscillations and their developmental trajectory in humans in the early years of life remains poorly understood. This study is the first to capitalize on the potential of OPM-MEG for the investigation of gamma oscillations from toddlerhood to adulthood, and the first to apply a DCM to OPM data to explore the underpinnings of gamma signals.

Using a well-established visual paradigm, we showed that age has a significant impact on the spectro-temporal neurophysiological response from the visual cortex. In the broadband gamma frequency range (30–80 Hz), low-amplitude oscillations are present, even early in childhood, and appear to remain through to adulthood. However, in later childhood we see a multi-spectral response, with a higher frequency (>60 Hz) component that agrees with the previous literature in school-aged children (Gaetz et al., 2011;Orekhova et al., 2018,^2023^) and a lower frequency component (~30 Hz) that falls into the high beta band. These are then followed by the well-established higher-amplitude band limited oscillations (at ~50–60 Hz) which are present in adulthood, and thus agreeing with previous studies (Hoogenboom et al., 2006;Muthukumaraswamy et al., 2010). Statistical analyses showed a significant increase in oscillatory amplitude with age in frequency bands spanning 45–65 Hz, with a peak change in the 51–55 Hz window. It is worth noting that the PSDs during stimulation and rest (Fig. S2) show these signals are not driven by changes in the aperiodic slope, which has been shown to flatten with age and be implicated in E-I balance (Gao et al., 2017;Vandewouw et al., 2024). Despite these significant spectral changes, we saw no measurable shift in the spatial origin of gamma oscillations with age, with the maximum signal consistently localised to the primary visual cortex.

Our results also highlight that visual gamma, even in adults, has high inter-individual differences and this agrees with other reports employing similar paradigms (e.g.,Muthukumaraswamy et al., 2010). This lack of consistency of strong-induced gamma oscillations across individuals may be due to paradigm or system design. Despite evidence that OPM systems could be more sensitive than conventional MEG systems, our system was not optimised specifically for the detection of these signals; it was structured for whole-head uniform coverage. Future work should investigate whether an optimised system design (i.e., dense coverage of triaxial OPM sensors across visual cortices (e.g., inHill et al., 2024)) may improve capture of induced gamma signal from younger participants. Further, while our visual paradigm was clearly able to induce visual gamma oscillations from our participants, previous studies in school aged children typically employed a larger stimulus and more trials (Orekhova et al., 2018); our lower amplitude signals may, therefore, be due in part to stimulation parameters. Further work should investigate the optimal stimulus to robustly induce gamma oscillations across the lifespan.

Despite a lower amplitude gamma response in children, the suppression of alpha oscillatory amplitude during visual stimulation was relatively stable across all age groups. In the 9–13 Hz band, alpha suppression showed no significant relationship with age; this provides a key validation of data quality across our dataset (i.e., if data were of poorer quality in younger participants, we would likely see a drop in alpha suppression in those individuals, which is not the case). We did, however, see a trend towards increased 5–9 Hz modulation in younger participants and increased 11–15 Hz modulation in adults. This is in good agreement with other studies (Miskovic et al., 2015) which show a shift in alpha peak frequency with age (albeit typically in resting state data), with younger subjects tending to have a lower alpha frequency. We further confirmed this by directly testing peak alpha frequency during the rest period, showing a significant increase with age inFigure S3. This provides further verification of our data quality.

Our DCM illustrates how age-related changes in gamma oscillations are driven by a neural circuit that matures with age. Specifically, our results show that several parameters demonstrate an age dependency: excitatory signals from spiny stellate cells to inhibitory interneurons (parameter G5) are significantly increased, and the relative excitatory versus inhibitory signalling from superficial pyramidal neurons to inhibitory interneurons (the ratio of parameters G12 and G11) is significantly decreased in adults compared to children. Previous work has demonstrated that G5 relates to beta and gamma amplitudes (Shaw et al., 2017); thus, this is in strong agreement with our spectral results, where we showed increased gamma amplitude in older participants. A decrease in the ratio between G12 and G11 supports our initial hypothesis that maturation would see a change in E-I balance (Larsen et al., 2022), such that inhibition in the superficial layer of the visual cortex increases, while excitation decreases, with age. This is likely due to an increase in gamma aminobutyric acid (GABA) (Jansen et al., 2010) and a relative decrease in glutamate (Hädel et al., 2013). We are the first to implicate these age-related changes via assessment of visual gamma oscillations. It is important to note that the model used is a simplified approach to infer the biophysical origin of such signals, and we have necessarily assumed that the structure of the model is consistent throughout development (we only consider the relative strength of connections to vary through age). This is supported, however, by the fact that the laminar composition of the cortex is formed during early gestation (Terashima et al., 2021).

A variety of methods have been used previously to investigate E-I balance and the development of excitatory and inhibitory signalling in early life. In animal models, invasive electrophysiological techniques allow direct measurement of synaptic inputs and neural firing. For example, studies on the early postnatal development of mice showed maturation of inhibitory signalling in the somatosensory cortex led to a rapid developmental decrease in E-I ratio (Zhang et al., 2011). Optogenetic stimulation has enabled direct manipulation of E-I balance in mice models, demonstrating the developmental tilt of E-I balance towards inhibition (Chini et al., 2022). In humans, functional MRI and magnetic resonance spectroscopy have been used to infer E-I balance through metabolic activity and neurotransmitter concentrations (Larsen et al., 2022;McKeon et al., 2024). However, these measures suffer from low temporal resolution, reliance on indirect mechanisms, and a challenging scanning environment. For characterisation of the early development of E-I balance, OPM-MEG offers unique advantages, combining high temporal resolution and non-invasive measurement of neural signals directly related to excitatory and inhibitory signalling with a naturalistic scanning environment. These features position OPM-MEG as a powerful tool for bridging the gaps between human and animal studies of the development of E-I signalling.

This study provides an important foundational step in the measurement of E-I balance via gamma oscillations in neurodevelopment. However, there are limitations which should be addressed. Firstly, OPM-MEG systems remain a new technology; OPMs have a higher noise floor than conventional MEG sensors, and the number of measurement channels is lower (again compared to conventional MEG instrumentation). However, we did use helmets which are lightweight, allow subject movement, and come in multiple sizes enabling adaptation for age. This ameliorates confounds of SNR change with age and movement, which (anecdotally) was large in children. We believe this study would not have been possible using either conventional MEG (due to confounds of head size and movement) or EEG (due to gamma oscillations being obfuscated by muscle artefacts). Importantly, OPM systems are still under development, and it is highly likely that sensor density (Hill et al., 2024) and noise floor will improve with time, meaning OPM-MEG will likely become the technique of choice for high-fidelity characterisation of brain function in neurodevelopment in the future. Secondly, to increase participant numbers, data were collected from two sites, potentially introducing a confounding effect of scanner configuration. To mitigate this, we matched recording conditions as far as possible, and a cross-site comparison within our adult groups (Fig. S1) showed no significant differences between sites. Further, at both sites we studied children and adults, meaning any measurable age-related differences are unlikely to be driven by site. We, therefore, think it is unlikely that our results could be affected by the cross-site nature of recordings; indeed, the fact that we were able to demonstrate cross-site reliability is extremely positive to accelerate the (already rapid) uptake of OPMs and to support the collection of new large, across-site datasets. A final limitation is that we have a non-uniform range of participant age; while this was enough to demonstrate significant age-related changes, the addition of adolescents and older adults to this study would enable elucidation of non-linear trajectories. Future work will aim to fill these gaps.

An imbalance in excitatory and inhibitory neurotransmission underlies current theories for the pathophysiological underpinnings of neurodevelopmental and psychiatric disorders. However, the study of these signals has been limited by technology, restricting most studies to adults, animal models, and the lab benchtop. OPM-MEG lifts these constraints, allowing us to measure signals relating to E-I balance directly, and from early life. We have demonstrated this important milestone and our results—which show significant changes in gamma oscillations and E-I balance with age—offer insight into early cortical maturation and provide a typically developing standard, from which clinical applications can be explored.

## Supplementary Material

Supplementary Material

## Data Availability

Data from UoN will be made available on Zenodo. Data from SickKids will be available through Ontario Brain Institute. OPM analysis code will be made available on GitHub (https://github.com/nsrhodes/gamma_opm_2024). Dynamic causal modelling was performed using a variant of DCM-SSR in SPM8, and code will be made available upon request.
