# Supplementary material for: Measuring the neurodevelopmental trajectory of excitatory-inhibitory balance via visual gamma oscillations
Source: Imaging Neurosci (Camb). 2025 Apr 6;3:imag_a_00527. doi: 10.1162/imag_a_00527 (PMC12319984; doi:10.1162/imag_a_00527)
Supplement: Supplementary Material [file imag_a_00527-supp.pdf]

Supplementary information for

Measuring the neurodevelopmental trajectory of excitatory-inhibitory balance via visual gamma oscillations

Natalie Rhodes[1,2,3*], Lukas Rier[1,4], Krish D. Singh[5], Julie Sato[2,3], Marlee M. Vandewouw[2,3,6], Niall Holmes[1,4], Elena Boto[1,4], Ryan M. Hill[1,4], Molly Rea[4], Margot J. Taylor[2,3,7+] and Matthew J. Brookes[1,4+]

[1] Sir Peter Mansfield Imaging Centre, School of Physics and Astronomy, University of Nottingham, Nottingham, NG7 2RD, UK
[2] Diagnostic Interventional Radiology, The Hospital for Sick Children, 555 University Avenue, Toronto, M5G 1X8, Canada
[3] Program in Neurosciences & Mental Health, SickKids Research Institute, 686 Bay Street, Toronto, M5G 0A4, Canada
[4] Cerca Magnetics Ltd., 2 Castle Bridge Road, Nottingham, NG7 1LD, UK
[5] Cardiff University Brain Research Imaging Centre, School of Psychology, Maindy Road, Cardiff, CF24 4HQ, UK
[6] Autism Research Centre, Bloorview Research Institute, Holland Bloorview Kids Rehabilitation Hospital, 150 Kilgour Road, Toronto, M4G 1R8, Canada
[7] Department of Medical Imaging, University of Toronto, 263 McCaul St, Toronto, M5T 1W7, Canada

+ denotes equal contribution

* denotes corresponding author:

Dr. Natalie Rhodes
The Hospital for Sick Children
555 University Avenue
Toronto,
Ontario
Canada
M5G 1X8
Email: natalie.rhodes@sickkids.ca

Keywords: gamma oscillations, excitation/inhibition balance, neurodevelopment, magnetoencephalography, optically pumped magnetometers.

Supplementary Information Text

Additional Methods

Site comparison:

A table describing the differences between the two systems is shown in Table. S1.

	**UoN**	**SickKids**
**No. sensors**	64	40
**Measurement axes per sensor**	3	2
**No. channels**	192	80
**Helmet**	Rigid 3D printed helmet - three sizes were used (adult S, adult L, 4-year-old)	Rigid 3D printed helmet - two sizes were used (adult L, 4-year-old)
**Magnetic environment**	Quiet university campus location outside the main city centre	Downtown location above a busy car park near construction sites, main roads and underground transport lines
**Passive shielding**	OPM-optimised shielded room with four layers of mu-metal and one layer of copper	Repurposed cryogenic MEG room with two layers of mu-metal and one layer of aluminium
**Degaussing coils**	Yes	No
**Typical magnetic environment with only passive shielding**	~ 2 nT static magnetic field with drifts of $\pm$ 1 nT in 10 minutes **(Rea et al., 2021)**	~ 30 nT static magnetic field with drifts of $\pm$ 20 nT in 10 minutes **(Hill et al., 2022, p. 20)**
**Active shielding (static)**	Via average coil currents through biplanar coils	Via reference array through biplanar coils
**Active shielding (dynamic)**	Not required (due to minimal field drift)	Via reference array through biplanar coils

Table S1. Site comparison. *A table describing the features of the OPM-MEG systems at each site.*

There are three main differences between the systems. First, the UoN system comprised 64 triaxial OPMs (QuSpin Inc. Triaxial Gen-3), providing 192 channels of data. The SickKids system used 40 dual axis OPMs (QuSpin Inc. Dual-axis Gen-3), providing 80 channels of data. The radial axis (available with both Dual- and tri-axial OPMs) provides the largest MEG signal (Iivanainen et al., 2017; Sarvas, 1987). Second, the magnetic

environment at each site differs, with a contrast between a city-centre lab and a green campus-based lab, as described by Hill et al. (Hill et al., 2022). Finally, the magnetic shielding between systems is different, with UoN benefitting from OPM-optimized shielding whereas the SickKids system uses a re-purposed room from a cryogenic MEG system. Despite these contrasting sites, we found no significant differences in visual gamma response between our age- and sex-matched groups of healthy adults (see Figure S1). This builds upon the result from a previous study with an individual participant (Hill et al., 2022), extending the comparison to consider group analyses.

The "Faces Circles" paradigm: Figure 1 b) shows an example of the circular grating and it is this that induces gamma oscillations which are the topic of investigation for the paper. However, the paradigm, also included images of emotional faces, with "faces" trials interspersed between circles trials. Additional cartoon character "catch trials" were also used to maintain attention from young participants. However, neither the faces nor the catch trials were analyzed here.

Each trial started with either an emotional face image, presented for 500 ms, or an inward drifting circular grating (oscillating at 1.2 $°\boldsymbol{s}^{-1}$) presented for 1 s. Following this, there was a rest period in which a white fixation cross located centrally on a black screen was presented for a randomly jittered period between 1050 – 1450 ms. There was a total of 60 circles trials and between 80 – 120 faces trials. The total experiment lasted between 5-8 minutes.

Participant demographics: Table S1 provides a detailed description of the demographics from each group of participants, separated into UoN and SickKids adults and children. One child was excluded from analyses due to the inability to acquire a 3D digitization for coregistration. In addition, one adult participant scan from SickKids was excluded from the main text analyses as that individual had also been scanned in Nottingham. The repeat scan was included in the cross-site comparison in Figure S1.

	**No. scans**	**Age (years, mean +/- standard deviation)**	**Age range (years)**	**No. males**	**No. females**
**UoN Children**	27	7.67 ± 3.09	2 – 13	10	17
**UoN Adults**	26	26.35 ± 3.29	21 – 34	12	14
**SickKids Children**	24	3.79 ± 0.76	3 – 5	10	14
**SickKids Adults**	26	26.27 ± 3.26	21 – 34	12	14
Total	103	16.17 ± 10.71	2 – 34	44	59

Table S2. *Participant demographics. A table describing the demographics of the participants scanned in the study across the two sites and age group cohorts*

Additional Results

Cross-site comparison: Figure S1 shows a comparison of gamma measurements acquired at the two sites. Data are shown for two age and sex-matched adult groups. Figure S1a shows the mean and standard deviation of the locations of the peak change in gamma modulation; these locations have been derived for each participant, projected to an average brain, and are shown as ellipsoids; the axes of the ellipsoid represent the standard deviation of the peak location coordinates in x, y and z; the centre of the ellipsoid represents the mean locations. We show results for three cases; UoN all channels; SK all (80) channels and UoN 80 channels; the latter is included to ensure equivalence in terms of channel count across sites. We found no significant differences between peak locations, suggesting that the two systems were equivalent in terms of delineation of the spatial signature of gamma modulation.

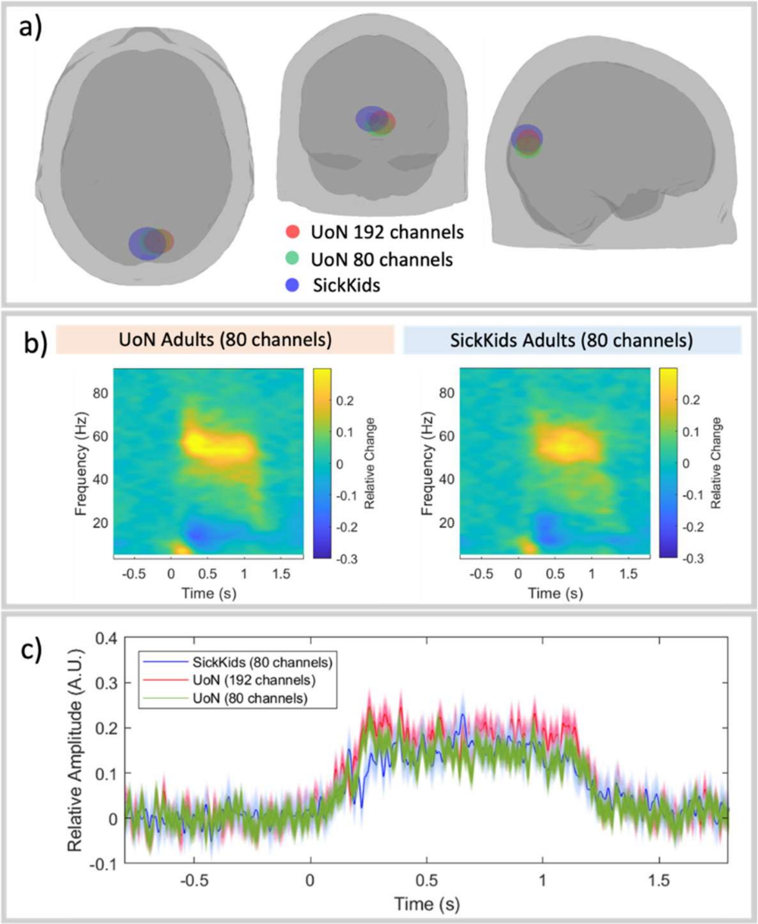

Figure S1. Cross-site comparison in a group of adults. *a) The mean and standard deviation of peak locations for gamma modulation. The centre of the ellipsoids represents the mean location of maxima; the ellipsoid axes represent standard deviation in the x, y and z coordinates. The UoN analyses was performed for both the full channel count and a reduced channel count (to 80) to match the SK system. b) The time-frequency spectrograms extracted from virtual electrodes at the peak of gamma modulation. c) Gamma band envelopes averaged across groups at SK and UoN, again with the UoN analyses performed for both the full channel count and reduced channel count.*

Figure S1b shows the group averaged time frequency spectra (TFS) for participants scanned at UoN (left) and SickKids (right). In both cases, the TFS is derived from a virtual electrode at the location of maximum gamma modulation; blue represents a decrease in oscillatory amplitude relative to baseline, yellow an increase. Time zero represents stimulus onset, and here, the UoN system has been reduced to 80 channels for comparison with SK. As expected, we see large increases in gamma activity with concomitant decreases in alpha and beta oscillations. Most importantly, activity at both sites looks similar. Figure S1c shows the envelope of 30 – 80 Hz oscillatory amplitude for the UoN system with full channel count (192 channels) in red, UoN reduced channel count in green and SickKids system in blue. A statistical analysis showed no significant differences between systems when measuring relative change in amplitude (measured between active ($0.3\ s \leq t \leq 1\ s$) and baseline ($-0.8 \leq t \leq -0.1\ s$) windows; $T = 1.01$, $p = 0.32$ for the full UoN channel count; $T = -0.26$, $p = 0.80$ for the reduced channel count). These data suggest that there is no measurable difference between sites when assessing the temporal modulation of gamma activity.

Power spectra: Figure S2 contrasts power spectra derived from z-scored data recorded during visual stimulation (0.3 to 1 s) and during the pre-stimulus rest period (-0.8 to -0.1 s) from the location of peak gamma oscillations. Spectra were computed using Welch's method with overlapping windows of 0.7 s. Each spectrum shows an increase in broadband gamma power during stimulation, with more narrowband gamma evident in the adult groups. This is in agreement with the results in the main manuscript.

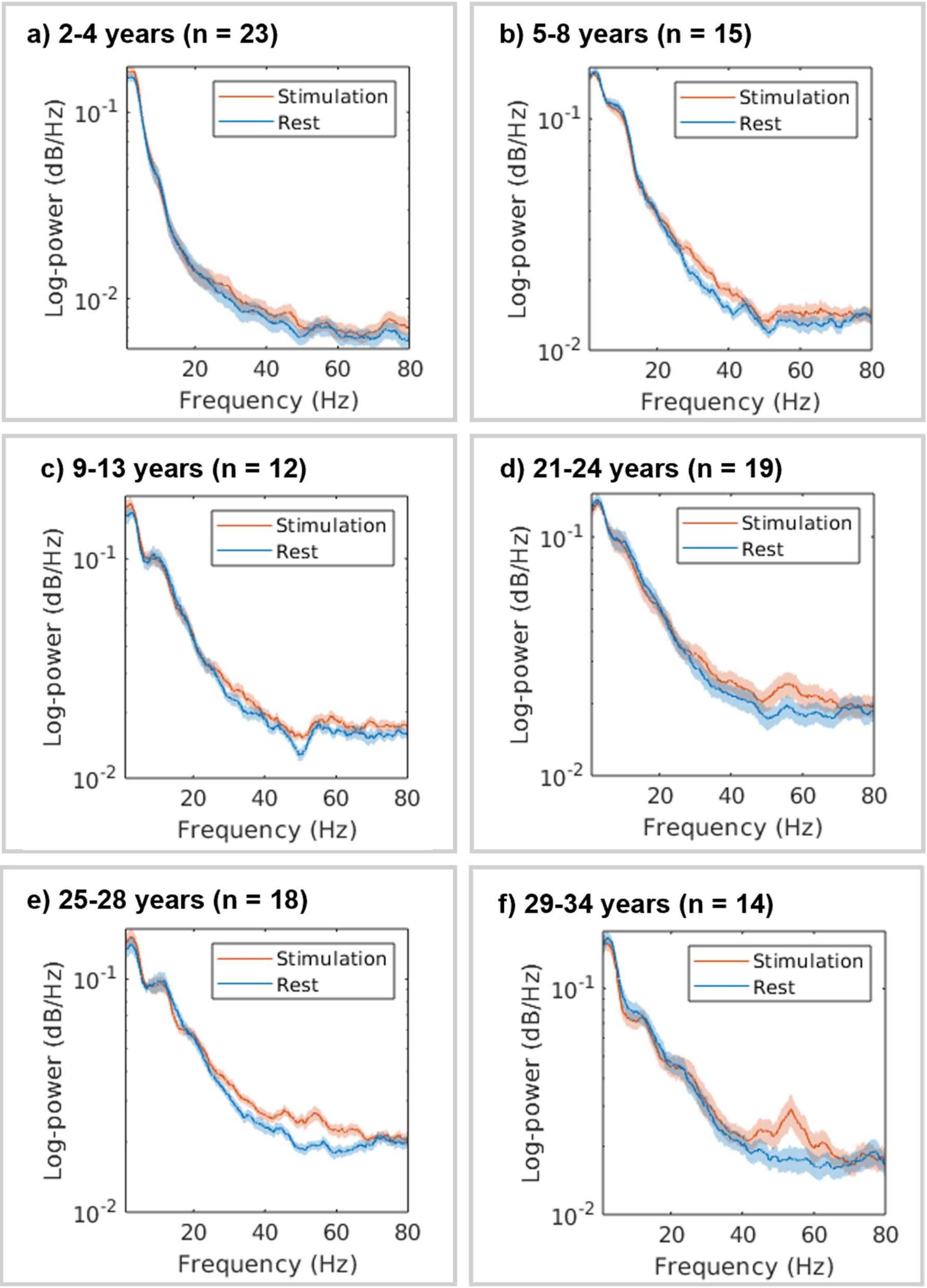

Figure S2. Stimulus and rest PSDs. *Power spectra acquired during stimulation and rest for a) 2–4-year-olds, b) 5-8 year olds, c) 9-13 year olds, d) 21-24 year olds e) 25-28 year olds f) 29-34 year olds. Shaded area represents standard error on the mean.*

Peak alpha frequency: We assessed peak alpha frequency from the virtual electrode at the location of peak alpha suppression. The PSD was computed using concatenated data from all trials during the rest period (i.e. no visual stimulus). This maximised alpha signal-to-noise ratio by avoiding the suppressive effects of the visual stimulus.

Peak alpha frequency was calculated using the spectral parametrization ('FOOOF') package in Python to separate the aperiodic activity (Donoghue et al., 2020). If FOOOF did not identify a peak in a broadened alpha range (6 – 13 Hz), the participant was not included in these analyses (n = 22). If multiple peaks were identified (n = 29), the peak with the largest amplitude was selected. Significance was assessed using Spearman's correlation, with the results shown in Figure S3. As shown, the peak alpha frequency increases significantly with age. This is driven by the child group, where separately they show a significant relation with age (slope = 0.40, p = 0.018), whilst the adult group showed no separate age relation (slope = -0.09, p = 0.58), as assessed by Spearman's correlation. This was expected as we know the alpha peak frequency typically increases in childhood and then remains somewhat stable through adulthood (Freschl et al., 2022).

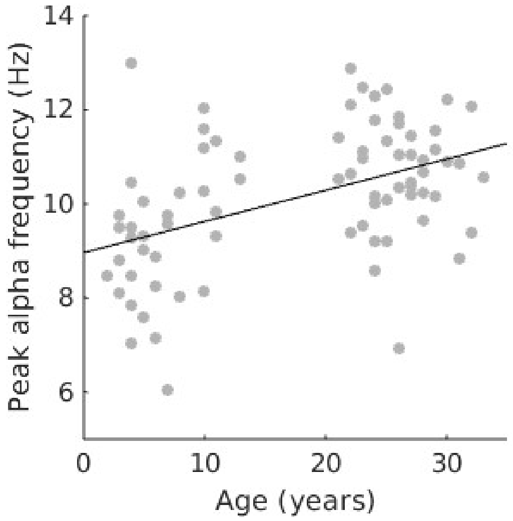

Figure S3. Peak alpha frequency with age. *Peak alpha frequency increases with age (slope = 0.45, p = 3.8×10^{-5}).*

Complete statistics from DCM:

Figure S4 shows the effect of age on the DCM model fit (as measured by the F-statistic, which represents the log model evidence, a measure of model fit with a complexity penalty). The relation between the F statistic and age was assessed using Spearman's correlation, finding no significant effect of age (r = -0.01, p = 0.92). This suggests that age-related differences observed in the model parameters are not driven by the quality of fit of the DCM.

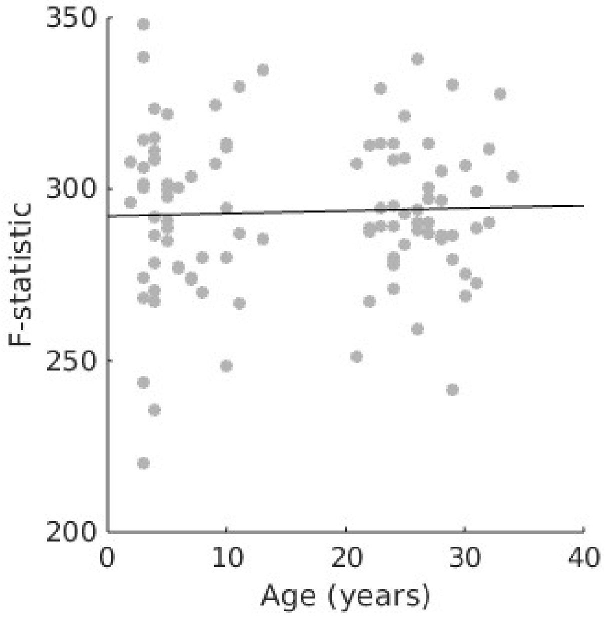

Figure S4. DCM model fit (F-statistic) with age. *There is no significant effect of age on the goodness of the model fit (r = -0.01, p = 0.92) measured by Spearman's correlation.*

Table S3 provides the Pearson correlation and p-values for the relation between the spectral parameters of the data and the corresponding G parameters from the model. These results are for direct comparison with the previous application of this same model in Shaw et al. 2017, which used conventional MEG to investigate the model outputs with adult participants in a similar visual task. We find very similar relations between G parameters and spectral features as those described in Table 2 of Shaw et al. 2017. Briefly, these include the positive association between parameter G5 and G8 with beta and gamma amplitudes, G6 and beta frequency and amplitude, G9 with beta amplitude and G11 with gamma amplitude. We also found a negative association between G11 and gamma frequency.

	G4	G5	G6	G7	G8	G9	G11	G12
**Beta frequency**	0.08 [0.4059]	-0.08 [0.4329]	0.41 [2.6e-5]	0.05 [0.6817]	0.14 [0.1649]	0.00 [0.9905]	0.16 [0.1168]	0.15 [0.1250]
**Beta amplitude**	-0.03 [0.7829]	0.40 [3.7e-5]	0.42 [1.3e-5]	0.08 [0.4067]	0.44 [5.8e-6]	0.47 [8.6e-7]	0.29 [0.0031]	0.19 [0.0614]
**Gamma frequency**	0.03 [0.7935]	-0.26 [0.0091]	-0.04 [0.6852]	0.19 [0.0625]	-0.27 [0.0072]	0.05 [0.6109]	-0.36 [2.6e-4]	-0.02 [0.8735]
**Gamma amplitude**	0.02 [0.8426]	0.66 [4.8e-14]	0.17 [0.0876]	-0.05 [0.6506]	0.58 [1.9e-10]	-0.13 [0.1824]	0.49 [2.0e -7]	-0.16 [0.1082]

Table S3. G parameters and MEG features. *A table outlining the Pearson correlation [with p-value] between spectral features of the MEG signal and the model parameters. Significant relations are highlighted in red (positive) and blue (negative). The threshold for significance is corrected for multiple comparisons using Bonferroni correction and set as 0.0016 (32 comparisons).*

Table S4 provides the summary of the statistics from the DCM describing the relations between model parameters and age, while covarying for sex.

	G4	G5	G6	G7	G8	G9	G11	G12	G6/G9	G12/G11
**R**	0.10	0.39	-0.13	-0.11	0.22	-0.19	0.28	-0.22	0.14	-0.43
**p value**	0.32	6.6e-5	0.20	0.28	0.03	0.05	0.005	0.03	0.17	8.7e-6

Table S4. G parameter correlation with age. *Statistical analysis of the relation of model outputs with age using Spearman's correlation. The threshold for significance is corrected for multiple comparisons using Bonferroni correction and set as 0.005 (10 comparisons).*

Sex effects: We re-ran the statistical analyses separately for the G12/G11 ratio to investigate sex-related effects and showed the same relation for both male and female participants. The data are shown in Figure S5.

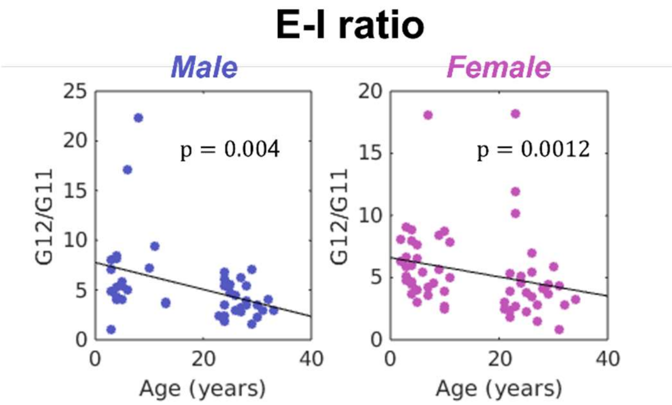

Figure S5. E-I ratio for male and females. *Parameter G12/G11 against age for male participants (left) and female participants (right).*

References

Donoghue, T., Haller, M., Peterson, E. J., Varma, P., Sebastian, P., Gao, R., Noto, T., Lara, A. H., Wallis, J. D., Knight, R. T., Shestyuk, A., & Voytek, B. (2020). Parameterizing neural power spectra into periodic and aperiodic components. *Nature Neuroscience*, *23*(12), 1655–1665. https://doi.org/10.1038/s41593-020-00744-x

Freschl, J., Azizi, L. A., Balboa, L., Kaldy, Z., & Blaser, E. (2022). The development of peak alpha frequency from infancy to adolescence and its role in visual temporal processing: A meta-analysis. *Developmental Cognitive Neuroscience*, *57*, 101146. https://doi.org/10.1016/j.dcn.2022.101146

Hill, R. M., Devasagayam, J., Holmes, N., Boto, E., Shah, V., Osborne, J., Safar, K., Worcester, F., Mariani, C., & Dawson, E. (2022). Using OPM-MEG in contrasting magnetic environments. *NeuroImage*, *253*, 119084.

Iivanainen, J., Stenroos, M., & Parkkonen, L. (2017). Measuring MEG closer to the brain: Performance of on-scalp sensor arrays. *Neuroimage*, *147*, 542–553.

Rea, M., Holmes, N., Hill, R. M., Boto, E., Leggett, J., Edwards, L. J., Woolger, D., Dawson, E., Shah, V., & Osborne, J. (2021). Precision magnetic field modelling and control for wearable magnetoencephalography. *NeuroImage*, 118401.

Sarvas, J. (1987). Basic mathematical and electromagnetic concepts of the biomagnetic inverse problem. *Physics in Medicine and Biology*, *32*(1), 11–22.
